# CD24 regulates cancer stem cell (CSC)-like traits and a panel of CSC-related molecules serves as a non-invasive urinary biomarker for the detection of bladder cancer

**DOI:** 10.1038/s41416-018-0291-7

**Published:** 2018-10-17

**Authors:** Akira Ooki, Christopher J. VandenBussche, Max Kates, Noah M. Hahn, Andres Matoso, David J. McConkey, Trinity J. Bivalacqua, Mohammad Obaidul Hoque

**Affiliations:** 1 Department of Otolaryngology-Head and Neck Surgery, The Johns Hopkins University School of Medicine, Baltimore, MD 21231 USA; 2 Department of Pathology, The Johns Hopkins University School of Medicine, Baltimore, MD 21231-2410 USA; 3 Department of Urology, The Johns Hopkins University School of Medicine, Baltimore, MD 21287 USA; 4 Department of Oncology, The Johns Hopkins University School of Medicine, Baltimore, MD 21231 USA

**Keywords:** Bladder cancer, Tumour biomarkers, Cancer stem cells

## Abstract

**Background:**

CD24 is a cornerstone of tumour progression in urothelial carcinoma of the bladder (UCB). However, its contribution to cancer stem cell (CSC)-like traits and the clinical utility of CD24 as a urinary biomarker for cancer detection have not been determined.

**Methods:**

The functional relevance of CD24 was evaluated using in vitro and in vivo approaches. The clinical utility of CSC-related molecules was assessed in urine samples by quantitative RT-PCR.

**Results:**

The knockdown of CD24 attenuated cancer stemness properties. The high-CD24-expressing cells, isolated from patient-derived UCB xenograft tumours, exhibited their enhanced stemness properties. CD24 was overexpressed not only in primary tumours but also in urine from UCB subjects. By assessment of 15 candidate CSC-related molecules in urine samples of a training cohort, a panel of three molecules (CD24, CD49f, and NANOG) was selected. The combination of these three molecules yielded a sensitivity and specificity of 81.7% and 74.3%, respectively, in an independent cohort. A combined set of 84 cases and 207 controls provided a sensitivity and specificity of 82% and 76%, respectively.

**Conclusion:**

CD24 has a crucial role in maintaining the urothelial cancer stem-like traits and a panel of CSC-related molecules has potential as a urinary biomarker for non-invasive UCB detection.

## Introduction

Urothelial carcinoma of the bladder (UCB) is the most common malignancy of the urinary tract, with an estimated 79,030 new cases and 16,870 deaths from the disease per year in the United States.^1^ The 5-year relative survival rate is > 90% when detected as a non-muscle invasive bladder cancer (NMIBC), while it drops to < 50% for muscle invasive disease.^2^ Diagnosis at early stage of the initial and recurrent disease is crucial for favourable outcomes. However, the estimated recurrence rate of NMIBC is 60–70, and 10–30% of these patients will progress to muscle invasive bladder cancer (MIBC) despite curative intensive therapy.^3,4^ This high recurrence rate requires patients to undergo frequent and life-long monitoring. Although non-invasive, highly-specific urine cytology assay is commonly used for the surveillance of UCB patients, its sensitivity is relatively low, specifically for low-grade tumours.^5–8^ Clinically robust, sensitive, and specific urinary biomarkers are needed to supplement urine cytology test.

Tumours are hierarchically organized by a rare population of cancer stem cells (CSCs) that contributes to cancer initiation, progression, and treatment failure.^9,10^ A better understanding of the molecular mechanisms underlying urothelial CSC regulation and the identification of key molecules associated with CSC generation and maintenance are pivotal for the determination of biology-based accurate biomarkers for early cancer detection, monitoring following transurethral resection of the bladder tumor (TURBT), and molecular-targeting therapy. We recently demonstrated that CD24 is a crucial CSC marker that is overexpressed in urothelial CSCs.^11^ It was also reported previously that CD24 acts as a hub of tumorigenesis and metastatic progression, and associated with a poor outcome in UCB.^12–15^ Moreover, CD24 deficiency reduced urothelial tumorigenesis and metastasis in a mouse model,^16^ and treatment with an anti-CD24 monoclonal antibody resulted in a decreased metastatic tumour burden.^15^ Thus, CD24 has been implicated in tumour initiation and progression as an oncogene, and it is a potential therapeutic target for UCB. However, the oncogenic role of CD24 in UCB is incompletely understood. Although CD24 has been characterized as a major determinant of stemness in other cancer types, including liver^17^ and colorectal carcinoma,^18^ it is still unclear whether CD24 functionally contributes to urothelial CSC-like traits. Furthermore, although meta-analysis indicated that CD24 is an important marker of malignancy, including for UCB,^12^ its clinical utility as a biomarker for cancer detection has not been tested yet.

We recently demonstrated that chronic arsenic exposure endows urothelial cells with malignant stemness properties, including increased expression of several CSC-related molecules such as SOX2, CD24, and NANOG.^19^ Furthermore, we observed incremental expression of SOX2 in urine samples from carcinogen (arsenic)-exposed non-cancer subjects and UCB subjects compared with urine samples from non-exposed control subjects. Given these findings and the central role of CSCs at the top of the cellular hierarchy in tumour initiation, we hypothesized that urothelial CSC-related molecules may serve as urinary biomarkers for discriminating between subjects with and without UCB. In this study, we performed a quantitative expression assessment of 15 CSC-related molecules in urine samples from 24 non-cancer control and 24 UCB subjects to construct a candidate panel of urinary biomarker for UCB detection. After determining the analytical and clinical sensitivity of a panel of three genes (CD24, CD49f, and NANOG) in a set of primary tumours with the matched urine, we evaluated the clinical utility of this panel of three CSC-related molecules in an independent cohort of urine samples from 60 UCB and 183 control subjects. Furthermore, we evaluated the functional contribution of CD24 to urothelial cancer stem-like traits using in vitro and in vivo approaches.

## Materials and methods

### Cell lines and tissue samples

UCB cell line 5637 was obtained from the American Type Culture Collection (ATCC, Manassas, VA, USA). BFTC 905 and BFTC 909 cell lines were obtained from the German Collection of Microorganisms and Cell Cultures (Braunschweig, Germany). A total of 5637 cells were grown in RPMI 1640 medium and the other cells were grown in Dulbecco’s modified Eagle’s medium (DMEM). Re-authentication of cells was performed using PowerPlex 16 HS for short tandem repeats analysis at the Johns Hopkins University School of Medicine (JHUSOM), Institute of Genetic Medicine core facility, and all cell lines have been confirmed as authentic. Urine samples from a total 84 UCB subject (24 for training s and 60 for validation cohorts) and 207 from population-matched subjects (24 for training and 183 for validation cohort) were analysed. These urine samples were obtained from a urinary tract specimen bank maintained within the JHUSOM, Department of Pathology. Control subjects of both the cohorts had no history of genitourinary malignancy. Diagnosis of all UCB specimens was confirmed by a board-certified cytopathologist. Detailed clinicopathological information of UCB cases and controls are provided in Table 1. Thirty human primary UCB and the corresponding adjacent histologically non-cancer urothelial tissue samples were obtained from JHUSOM, Department of Pathology. Informed consent was obtained from the patients before sample collection. Approval to conduct research on human subjects was obtained from the JHUSOM institutional review boards. This study qualified for exemption under the U. S. Department of Health and Human Services policy for protection of human subjects [45 CFR 46.101(b)].

**Table 1:** The clinicopathological features of urine cohorts in this study

Samples	Training cohort		Validation cohort	
	Tumour	Control	Tumour	Control
Patients	(*n* = 24)	(*n* = 24)	(*n* = 60)	(*n* = 183)
Age (years)				
Mean + SEM (years)	71.29 ± 2.13	62.79 ± 2.56	62.87 ± 1.25	63.21 ± 1.19
Median (years)	71	64	63	66
Range (years)	54–90	26–81	46–83	21–92
Race				
White	17	15	25	128
Black	4	8	4	42
Others	2	1	0	13
Unknown	1	0	31	0
Gender				
Female	7	6	15	55
Male	17	18	39	128
Unknown	0	0	6	0
Histological grade				
High	17	NA	38	NA
Low	6	NA	15	NA
Unknown	1	NA	7	NA
Invasion of the muscularis propria				
NMIBC	14	NA	47	NA
MIBC	0	NA	9	NA
Unknown	10	NA	4	NA
Cytology				
Negative^a^	16	NA	17	NA
Positive	8	NA	40	NA
Unknown	0	NA	3	NA

*MIBC* muscle invasive bladder cancer, *NA* not applicable, *NMIBC* non-muscle invasive bladder cancer

^a^Negative cytology includes atypical urothelial cells and suspicious urothelial cancer cells

### RNA extraction and quantitative reverse-transcriptase PCR

Total RNA from cell lines and formaldehyde-fixed paraffin-embedded human tissues was isolated using the RNeasy Plus Mini Kit (Qiagen, Valencia, USA) and the RecoverAll™ Total Nucleic Acid Isolation Kit (Ambion, Austin, USA), respectively. Urine samples were centrifuged for 5 min at 1500 r.p.m. and the supernatant was used for RNA extraction as described previously.^19^ Total RNA extraction from urine was performed using the MirVana miRNA Isolation Kit (Ambion). Quantitative reverse-transcriptase PCR (qRT-PCR) was performed using the Fast SYBR Green Master Mix (Thermo Fisher Scientific, Waltham, USA) on a 7900HT Fast Real-Time PCR System (Life Technologies, Carlsbad, USA) in triplicate. Primer sequences and the thermal cycling conditions were shown in Supplementary Table S1. SDS software (Applied Biosystems) was used to determine cycle threshold (Ct) values. Expression levels were quantified relative to β-actin using the 2^−ΔΔCt^ method.

### Candidate gene selection to evaluate as a urinary biomarker

To construct a panel of urinary biomarker for cancer detection, 15 potential CSC-related molecules were selected based on our previous findings associated with malignant stemness properties in UCB.^11,19^ A receiver operating characteristic (ROC) analysis was used for evaluating the UCB detection accuracy using urine. ROC analysis method circumvents fluctuations caused by the arbitrarily chosen cut-off value of expression level to differentiate cases and controls as a selection criteria. The optimal cut-off value for distinguishing between UCB and control urine samples was determined using the ROC analysis for each gene. The performance of ROC analysis for each gene was evaluated by the area under the curve that is a combined measure of sensitivity and specificity. In addition, the positive and negative likelihood ratio, which are not affected by the prevalence of the disease, were measured to assess the strength of UCB detection accuracy for each gene.

### The Cancer Genome Atlas analysis

The gene expression data of 19 primary UCB samples and the matched tumour adjacent histologically normal samples in the The Cancer Genome Atlas (TCGA) cohort^20^ was downloaded from the MethHC database^21^ to determine the expression level of our gene of interest in this external dataset.

### Western blotting analysis

Whol-cell lysates were extracted using the RIPA buffer (Thermo Scientific) supplemented with 10 μL/mL of the Halt™ Protease Inhibitor Cocktail Kit (Life Technologies) and 30 μL/mL of the Halt™ Phosphatase Inhibitor Cocktail Kit (Life Technologies). CD133 (A3G6K) and ATP-binding cassette subfamily G member 2 (ABCG2) (42078) antibodies were obtained from Cell Signaling Technology (Danvers, MA, USA). Yes-associated protein1 (YAP1) (ab52771) and CD24 (AF5247-SP) were obtained from Abcam (Cambridge, USA) and R&D Systems (Minneapolis USA), respectively. β-Actin (A2228) was obtained from Sigma-Aldrich (St. Louis, USA). Secondary horseradish peroxidase (HRP)-conjugated antibodies were obtained from Cell Signaling Technology. Chemiluminescent detection of HRP-labelled antibodies was performed using Amersham ECL Prime Western Blotting Detection Reagent (GE Healthcare, Piscataway, USA). Expression levels of all candidates were quantified by myImageAnalysis™ Software (Thermo Scientific) and normalized to β-actin.

### Gene silencing

CD24 short hairpin (shRNA) Lentiviral Particles (Cat # sc-29978-V) was used for the knockdown of the gene expression (CD24-sh; Santa Cruz Biotechnology, Dallas, USA). shRNA Lentiviral Particles (Cat # sc-108080) was used as a control (CD24-Ctrl; Santa Cruz Biotechnology). Cells were seeded in 24-well plates (5 × 10^4^ cells per well) for transduction. After 24 h, lentiviral particles were added to the cells in the presence of 8 mg/mL polybrene (EMD Millipore) and incubated at 37 °C for 4 h. The medium was then replaced with fresh medium. Stable cells harbouring CD24 shRNA were established by antibiotic selection and expression level of CD24 was confirmed by RT-PCR and western blotting in the respective clone.

### Sphere-formation assay and self-renewal assay

Sphere formation was performed by culturing cells (2 × 10^4^/well) in DMEM/Ham’s F12 50/50 Mix (Mediatech) supplemented with B-27 (Life Technologies), 20 ng/mL of fibroblast growth factors-basic (Peprotech, New Jersey, USA), and 20 ng/mL epidermal growth factor (Peprotech). Cell culture was performed in ultra-low attachment six-well plates (Corning, Lowell, USA) for 14 days. The medium was replaced every other day. Sphere formation was evaluated using the inverted phase-contrast microscope and single sphere with a diameter larger than 100 µm was counted using NIS-Elements Microscope Imaging Software (Nikon Instruments).

For the self-renewal assay, primary spheres were collected by gentle centrifugation (5 min at 400 × *g*), dissociated with Stempro Accutase Cell Dissociation Reagent (Life Technologies), and mechanically disrupted with a pipette. The cell suspension was sieved through 40 µm cell strainer cap filter to achieve a single-cell suspension. Equal numbers of live cells were plated in ultra-low attachment plates to generate the second spheres. All the experiments were performed in triplicate and repeated at least three times.

### Cell viability assay (MTT assay)

Cell viability was measured using the MTT (3-(4, 5-dimethyl thiazol-2-yl)-2, 5-diphenyltetrazolium bromide) Proliferation Assay Kit (ATCC, Manassas, VA, USA) according to the manufacturer’s protocol. Spheroid cells were treated with cisplatin (CDDP, Sigma-Aldrich) for 72 h in ultra-low attachment 96-well plates under serum-free condition. Each assay was performed in triplicate and each experiment was repeated at least three times.

### Invasion assay and wound-healing assay

The invasion assay was performed using the 24-well BD BioCoat Matrigel Invasion Chamber (BD Biosciences, San Jose, USA) as described previously.^22^ Cells that had invaded through the membrane were counted under a microscope in five randomly selected fields (magnification × 20) per well and averaged.

The wound-healing assay was performed using the Culture-Inserts (Ibidi, Verona, USA). The area of wound coverage was calculated using NIS-Elements Microscope Imaging Software.

### Flow cytometric analysis

For CD24 staining, cells (1 × 10^6^/100 μL stain buffer) were incubated with PE-conjugated anti-human CD24 antibody (cat #, 560991; BD Biosciences) for 30 min at 4 °C in dark. PE-conjugated IgG2a, κ-Isotype (cat #, 555574; BD Biosciences) was used as a control. Data were acquired on a BD FACSCalibur flow cytometer (BD Biosciences) using BD CellQuest Pro software (BD Biosciences).

For the apoptosis assay, the spheroid cells were exposed to CDDP (5 μM) for 72 h under serum-free medium and stained with PE Annexin V and 7-Amino-Actinomycin D (7-AAD) for discrimination of early and late apoptosis, respectively, using the Phycoerythrin (PE) Annexin V Apoptosis Detection Kit I (BD Biosciences).

### In vivo xenograft assay

NOD/SCID/IL2Rγ−/− (NSG) mice were obtained from the JHUSOM animal care facility. All experiments using mice were approved by the JHUSOM Animal Care and Use Committee, and mice were maintained under pathogen-free conditions within the JHUSOM animal care facility in accordance with the American Association of Laboratory Animal Care guidelines. For a limiting-dilution tumour formation assay, serially diluted CD24-sh or CD24-Ctrl spheroid cells (1 × 10^4^, 1 × 10^3^, or 1 × 10^2^ cells per flank) were suspended in 100 μL of a 1:1 mixture of serum-free DMEM and Cultrex Stem Cell Qualified Reduced Growth Factor Basement Membrane Extract (Trevigen, Gaitherburg, USA), and then injected subcutaneously into both flanks of 4–5 weeks old NSG mice. Tumour growth was monitored and tumour volume was calculated from caliper measurements of two orthogonal diameters [larger (*x*)] and smaller (*y*) diameters] using the following formula: volume = *xy*^2^/2. The mice were killed when tumour reached 2 cm in diameter or 70 days later.

Preserved patient-derived tumour xenograft (PDX) tissues (CTG1388 and CTG1061) were obtained from Champion Oncology (Maryland, USA). For magnetic-activated cell sorting for CD24, PDX tumours were minced and digested with collagenase type IV (Sigma-Aldrich), hyaluronidase (Sigma-Aldrich), and DNase type IV (Sigma-Aldrich) into Hank’s buffered salt solution, followed by depletion of red blood cells using ACK lysing buffer (Quality Biological, Gaithersburg, USA). Tumour cells were isolated using Tumour Cell Isolation Kit (Miltenyi Biotec) according to the manufacturer’s protocol. Then, tumour cells (1 × 10^8^) were labelled with PE-conjugated anti-human CD24 antibody (Miltenyi Biotec, Auburn, USA) and subsequently labelled with Anti-PE MultiSort MicroBeads (Miltenyi Biotech). After washing, separation for CD24-negative and -positive fraction was performed using MACS Columns and MidiMACS Separator (Miltenyi Biotec) twice. This process led to the separation of low-CD2 and high-CD24-enriched cell population. To confirm the separation, flow cytometric analysis was carried out using PE-conjugated anti-human CD24 antibody (Miltenyi Biotec). PE-conjugated anti-IgG1κ Isotype (Miltenyi Biotec) was used as controls for CD24 staining.

### Statistical analysis

In each set of data analyses, the estimate variation is indicated in each figure as a SEM. The two groups were compared with the Wilcoxon–Mann–Whitney test for continuous variables and the Fisher’s exact test for categorical variables, respectively. The level of statistical significance was set at *P* *<* 0.05. All statistical analyses were conducted using the JMP 12 software package (SAS Institute).

## Results

### Knockdown of CD24 attenuates urothelial cancer stemness properties

**Fig. 1:**
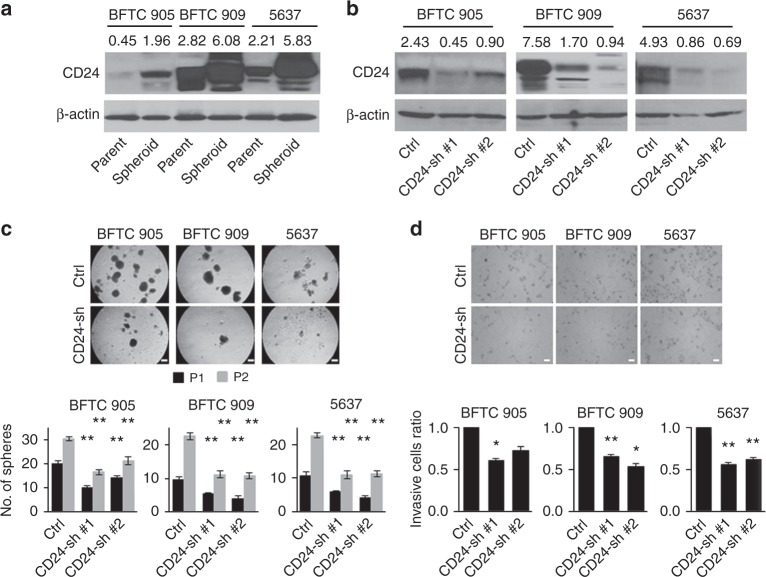
The sphere-forming and invasive abilities attenuated by CD24 knockdown in BFTC 905, BFTC 909, and 5637 cell lines. **a** Western blotting analysis of CD24 in isogeneic parental and spheroid cells. Upper, western blotting images; lower, expression levels of CD24 were quantified by myImageAnalysis™ Software and normalized to their respective β-actin. **b** Western blotting analysis of CD24 in stable CD24 knockdown (CD24-sh) and control (CD24-Ctrl) cells. Upper, western blotting images; lower, expression levels of CD24 were quantified relative to β-actin. **c** Sphere formation and self-renewal assays through the second (P2) passage from the first passage (P1) in stable CD24-sh cells compared with control CD24-Ctrl cells. Upper, representative images of sphere formation (scale bars, 200 μm); lower, number of the spheres over 100 µm. Data are from three independent experiments. **d** Invasion assay of spheroid CD24-sh cells. Upper, representative images (scale bars, 100 μm; lower, the relative number of invaded spheroid CD24-sh cells compared with spheroid CD24-Ctrl cells. Each error bar indicates mean ± SEM. *P < 0.05; **P < 0.01 (Wilcoxon–Mann–Whitney test)

To our knowledge, there is no report whether CD24 functionally contributes to urothelial cancer stem-like traits. As spheroid cells contain enriched stem cell populations,^23^ we first assessed the expression levels of CD24 in spheroid cells compared with the matched parental cells in BFTC 905, BFTC 909, and 5637 cell lines. The spheroid cells showed higher expression of CD24 than the matched parental cells (Fig. 1a). To determine the functional role of CD24 in urothelial CSCs, a lentiviral-based stable knockdown clones of CD24 (CD24-sh) were established in BFTC 905, BFTC 909, and 5637 cell lines (Fig. 1b). The effect of CD24 knockdown on sphere-forming and self-renewal abilities was analysed by sphere-formation assays. CD24-sh cells generated fewer and smaller spheres compared with the control (CD24-Ctrl) cells through their first and second passages (Fig. 1c). To assess whether spheroid CD24-sh cells sustain an aggressive phenotype comparable to the spheroid CD24-Ctrl cells, we performed invasion assays and found that spheroid CD24-sh cells demonstrated decreased invasion (Fig. 1d).

**Fig. 2:**
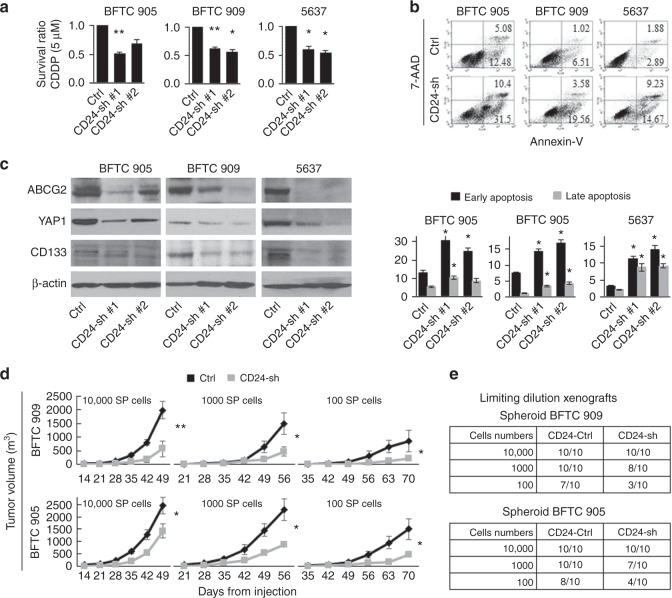
The chemoresistant and tumorigenic abilities attenuated by CD24 knockdown in BFTC 905, BFTC 909, and 5637 cell lines. **a** Cell viability after 5 μM cisplatin (CDDP) treatment for 72 h in the spheroid CD24-sh cells, as measured by MTT assay. Cell viability was expressed as the ratio of absorbance values of the spheroid CD24-sh cells related to the spheroid CD24-Ctrl cells considered as 1.0. Data are from three independent experiments. **b** An apoptosis assay of spheroid CD24-sh cells treated with 5 μM CDDP for 72 h. Upper, representative images of early apoptosis (bottom right quadrant) and late apoptosis (top right quadrant); lower, percentage of apoptotic cells. **c** Western blotting analysis of ABCG2, YAP1, and CD133 in stable CD24-sh and CD24-Ctrl cells. **d** Limiting-dilution xenograft assays in stable spheroid CD24-sh BFTC 909 (upper) and BFTC 905 (lower) cells. Tumour growth was measured after subcutaneous injection of serially diluted spheroid cells (1 × 10^4^, 1 × 10^3^, or 1 × 10^2^ cells per flank) into both flanks of NSG mice (five mice per group). SP, spheroid. **e** Tumour initiation frequency after the xenotransplantation of spheroid CD24-sh BFTC 909 (upper) and BFTC 905 (lower) cells. Tumour-initiating capacity is shown as the numbers of tumours/the number of injections after 70 days from subcutaneous injection of serially diluted spheroid CD24-sh cells (1 × 10^4^, 1 × 10^3^, or 1 × 10^2^ cells per flank) into both flanks of NSG mice (five mice per group). Each error bar indicates mean ± SEM. *P < 0.05; **P < 0.01 (Wilcoxon–Mann–Whitney test)

As CSCs are resistant to conventional chemotherapies that efficiently eliminate bulk tumour cells, the viability of spheroid CD24-sh cells was analysed by treating with cisplatin (CDDP), an important chemotherapeutic agent for the treatment of UCB. As expected, spheroid CD24-sh cells were more sensitive to CDDP treatment than spheroid CD24-Ctrl cells (Fig. 2a). Furthermore, we determined that CD24 knockdown significantly attenuated the anti-apoptotic ability against CDDP treatment in spheroid cells (Fig. 2b).

To determine the effect of CD24 knockdown on candidate CSC-related molecules, we tested mRNA expression levels of 15 potential CSC-related molecules in CD24-sh and CD24-Ctrl cells by qRT-PCR. CD24 knockdown led to the downregulation of numerous CSC-related molecules, among which CD133, YAP1, and the drug efflux transporter ABCG2 were found to be consistently altered due to loss of CD24 in the three UCB cell lines (Supplementary Fig. S1). We confirmed similar findings at the protein level (Fig. 2c).

### Knockdown of CD24 attenuates in vivo tumorigenicity

As an important feature of CSCs is efficient in vivo tumorigenesis at a limiting-dilution xenograft,^24^ we performed the tumour formation assays with serial dilutions of spheroid CD24-sh cells and CD24-Ctrl cells. Serially diluted spheroid CD24-sh or CD24-Ctrl cells (1 × 10^4^, 1 × 10^3^, or 1 × 10^2^ cells per flank) were injected subcutaneously into both flanks of NSG mice (five mice per group). Comparable with our in vitro assays data, the spheroid CD24-sh cells had significantly reduced tumour growth compared with the spheroid CD24-Ctrl cells (Fig. 2d). The spheroid CD24-Ctrl cells were sufficient for tumour development by injecting as few as 100 cells into NSG mice. Seven and eight tumours were developed in a total of ten flanks of five mice injected with spheroid CD24-Ctrl BFTC 909 and CD24-Ctrl BFTC 905 cells, respectively, within a 70-day follow-up period after cell injection. In contrast, the spheroid CD24-sh cells showed decreased tumour initiation frequency (development of three and four tumours per ten flanks of mice for CD24-sh BFTC 909 and CD24-sh BFTC 905 cells, respectively; Fig. 2e).

### CD24-expressing cells isolated from PDX models exhibit enhanced cancer stem-like traits

**Fig. 3:**
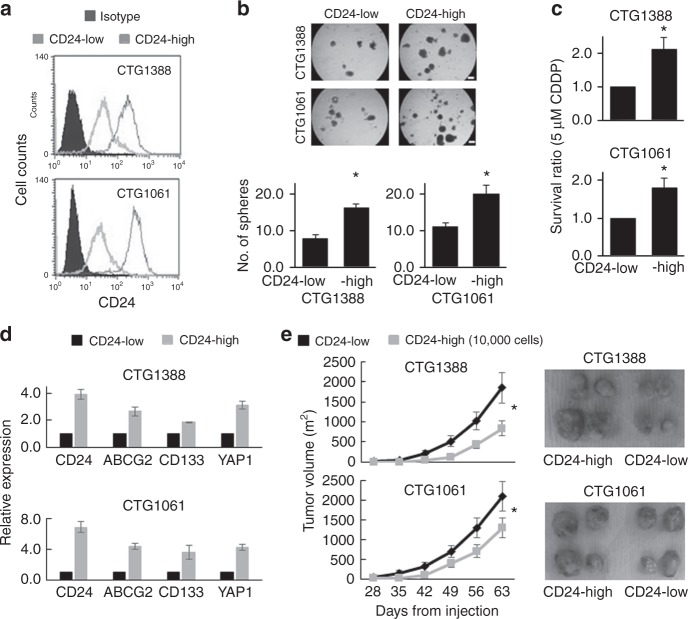
Enriched cancer stem-like traits in CD24-expressing cells. **a** Flow cytometry analysis after isolation of each subpopulation (high- and low-CD24-expressing cells) from PDX CTG1388 and CTG1061 tumours according to the endogenous status of CD24 expression using the magnetic-activated cell sorting. **b** Sphere formation assay after isolation of high- and low-CD24-expression cells. Upper, representative images of sphere formation (scale bars, 200 μm); lower, number of the spheres over 100 µm. Data are from three independent experiments. **c** Cell viability after 5 μM cisplatin (CDDP) treatment for 72 h in high- and low-CD24-expressing cells, as measured by MTT assay. Cell viability was expressed as the ratio of absorbance values of high-CD24-expressing cells treated with CDDP related to low-CD24-expressing cells considered as 1.0. Data are from three independent experiments. **d** Association of CD24 with ABCG2, CD133, and YAP1 expression in high-CD24-expressing cells compared with low-CD24-expressing cells. The relative mRNA expression levels of CD24, ABCG2, CD133, and YAP1 in high-CD24-expressing cells were calculated considering the expression values equal to 1.0 in low-CD24-expressing cells, as measured by qRT-PCR. **e** The in vivo tumorigenicity of xenotransplantation of high- and low-CD24-expressing CTG1388 (upper) and CTG1061 (lower) cells into both flanks of NSG mice (five mice per group). Left, tumour growth curve after subcutaneous injection of the cells (1 × 10^4^ cells per flank); right, representative images of tumours. Each error bar indicates mean ± SEM. *P < 0.05 (Wilcoxon–Mann–Whitney test)

To test whether cells with high endogenous CD24 expression possess enhanced stemness properties, we isolated high- and low-CD24-expressing cells from two PDX models using the magnetic-activated cell sorting approach. One model was established from a primary site (CTG1388) and a second model was established from a metastatic site (CTG1061). We confirmed the expression status of CD24 by flow cytometric analysis (Fig. 3a). The high-CD24-expressing cells exhibited greater sphere-forming and chemoresistant abilities than the low-CD24-expressing cells (Fig. 3b, c). In addition, the link of CD24 with CD133, YAP1, and ABCG2 was solidified by our observation of high expression of these molecules in PDX-derived high-CD24-expressing cells, as compared with low-CD24-expressing cells (Fig. 3d). Furthermore, the high-CD24-expressing cells grew faster and generated larger tumours than the low-CD24-expressing cells after subcutaneous injection of 1 × 10^4^ cells per flank into NSG mice (Fig. 3e). Collectively, our findings suggest a crucial role of CD24 in urothelial cancer stem-like traits.

### CD24 has potential as a urinary biomarker for UCB detection

**Fig. 4:**
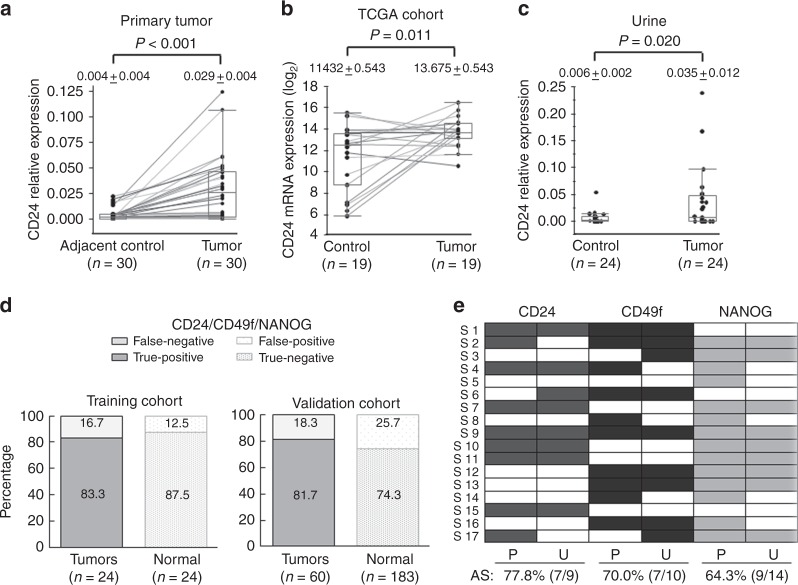
The cancer detection accuracy of a combination panel of three CSC-related molecules (CD24, CD49f, and NANOG) in urine samples. **a** Box plots of the relative expression levels of CD24 mRNA in 30 primary tumour and the matched adjacent normal tissues. Scatter plots show the distribution of individual expression value of CD24 determined by qRT-PCR. The expression levels of tumour and the matched adjacent tissues were connected with a line. **b** Box plots of the expression levels of CD24 mRNA in19 UCB samples and the matched adjacent, histologically normal samples in the TCGA cohort. The expression values (RSEM log_2_) of tumours and the matched adjacent tissues were connected with a line. **c** Box plots of the relative expression levels of CD24 mRNA in urine samples from 24 UCB and 24 control subjects. **d** Sensitivity and specificity of the combination panel for cancer detection in urine samples of the training cohort and independent validation cohort. The schematic representation shows true positives, false negatives, true negatives, and false positive detected by the combination panel of three molecules (CD24, CD49f, and NANOG). **e** Analytical sensitivity (AS) of CD24, CD49f, and NANOG in 17 primary UCB and the matched urine samples. Analytical sensitivity is defined as ‘The fraction of cases in which overexpression of a marker was found in urine RNA for case patients who had confirmed overexpression of the same marker in the primary tumour RNA’. Cells in colour represent the positive expression, defined by optimal cut-off value determined by ROC curve, for each molecule in primary tumour (P) and the matched urine (U) samples. Each data indicates mean ± SEM. The paired *t*-test (**a** and **b**) were performed

To test the cancer specificity of CD24 expression in primary UCB, we analysed mRNA expression levels of 30 primary UCB and matched adjacent histologically non-cancer tissues by qRT-PCR. Consistent with previous reports,^12–14^ CD24 showed significantly higher expression in primary tumours compared with the corresponding adjacent normal tissues (Fig. 4a). Furthermore, analysis of the TCGA UCB cohort generated similar findings in tumour and the matched adjacent normal tissues (Fig. 4b)

The clinical utility of CD24 as a biomarker for cancer detection has not been determined. Given the cancer-specific elevation of CD24 expression in primary tumours, we next assessed the potential for non-invasive cancer detection using a total of 48 urine samples (24 UCB and 24 control subjects) as a training cohort (Table 1). The expression level of CD24 in urine was significantly higher in UCB subjects than in controls (Fig. 4c). The optimal cut-off value for distinguishing between urine samples from UCB and control subjects was calculated using a ROC analysis. By using the optimal cut-off value of CD24 expression, the sensitivity and specificity of CD24 for cancer detection were 45.8% and 95.8%, respectively (Supplementary Table S2). The high specificity indicates that CD24 may be a potential urinary biomarker for UCB detection. We hypothesized that the low sensitivity was due to heterogeneity among UCB, and that the addition of other markers could improve sensitivity.

We previously reported SOX2 as a potential urine-based biomarker for non-invasive early detection of UCB,^19^ and this molecule is an established regulator of CSCs and have a considerable role in tumour initiation.^9^ To further identify CSC-associated urine-based biomarkers, we tested 15 CSC-related molecules by candidate gene approach in urine from 24 UCB and 24 controls subjects (total 48 urine samples as a training cohort). Among these 15 molecules, NANOG, CD49f, LGR5, ΔNp63, SOX2, and CD24 showed significantly higher expression levels in urine from UCB subjects compared with control samples (Supplementary Table S2). By determining the optimal cut-off using ROC curves for each molecule, the individual sensitivity and specificity of these six molecules (NANOG, CD49f, LGR5, ΔNp63, SOX2, and CD24) for cancer detection ranged from 29.2% to 62.5% and 83.3 to 100%, respectively (Supplementary Table S2). We further assessed the expression pattern spectrum of these six molecules. When the positive expression of at least one of the six molecules was considered, the sensitivity was 95.8%, whereas the specificity decreased to 50.0% (Supplementary Table S3). When combination of CD24, CD49f, and NANOG was considered, a high UCB detection accuracy was achieved, with a sensitivity of 83.3% and specificity of 87.5% (Fig. 4d and Table 2).

**Table 2:** The bladder cancer detection accuracy of a panel of three genes in urine samples

Characteristics	CD24		NANOG		CD49f		CD24/NANOG/CD49f	
	Sensitivity	Specificity	Sensitivity	Specificity	Sensitivity	Specificity	Sensitivity	Specificity
**Training cohort**	45.8% (11/24)	95.8% (23/24)	45.8% (11/24)	100% (24/24)	54.2% (13/24)	91.7% (22/24)	83.3% (20/24)	87.5% (21/24)
Tumour invasion								
NMIBC	21.4% (3/14)		57.1% (8/14)		64.3% (9/14)		78.6% (11/14)	
Grade of urothelial cancer								
Low	16.7% (1/6)		50.0% (3/6)		66.7% (4/6)		66.7% (4/6)	
High	58.8% (10/17)		47.1% (8/17)		52.9% (9/17)		94.1% (16/17)	
Cytology								
Negative^a^	43.8 (7/16)		50.0% (8/16)		56.3% (9/16)		81.3% (13/16)	
Positive	50.0% (4/8)		37.5% (3/8)		50.0% (4/8)		87.5% (7/8)	
	**Sensitivity**	**Specificity**	**Sensitivity**	**Specificity**	**Sensitivity**	**Specificity**	**Sensitivity**	**Specificity**
**Independent cohort**	35.0% (21/60)	91.3% (167/183)	51.7% (31/60)	88.5% (162/183)	35.0% (21/60)	83.6% (153/183)	81.7% (49/60)	74.3% (136/183)
Tumour invasion								
NMIBC	44.7% (21/47)		44.7% (21/47)		40.4% (19/47)		80.9% (38/47)	
Grade of urothelial cancer								
Low	40.0% (6/15)		33.3% (5/15)		33.3% (5/15)		80.0% (12/15)	
High	39.5% (15/38)		50.0% (19/38)		39.5% (15/38)		78.9% (30/38)	
Cytology								
Negative^a^	41.2% (7/17)		52.9% (9/17)		23.5% (4/17)		82.4% (14/17)	
Positive	35.0% (14/40)		47.5% (19/40)		40.0% (16/40)		80.0% (32/40)	
	**Sensitivity**	**Specificity**	**Sensitivity**	**Specificity**	**Sensitivity**	**Specificity**	**Sensitivity**	**Specificity**
**Combined cohort**	38.1% (32/84)	91.9% (190/207)	50.0% (42/84)	89.9% (186/207)	40.5% (34/84)	84.5% (175/207)	82.1% (69/84)	75.8% (157/207)
Tumour invasion								
NMIBC	39.3% (24/61)		47.5% (29/61)		45.9% (28/61)		80.3% (49/61)	
Grade of urothelial cancer								
Low	33.3% (7/21)		38.1% (8/21)		42.9% (9/21)		76.2% (16/21)	
High	45.6% (25/55)		49.1% (27/55)		43.6% (24/55)		83.6% (46/55)	
Cytology								
Negative^a^	42.4% (14/33)		51.5% (17/33)		39.4% (13/33)		81.8% (27/33)	
Positive	37.5% (18/48)		45.8% (22/48)		41.7% (20/48)		81.3% (39/48)	

*MIBC* muscle invasive bladder cancer, *NMIBC* non-muscle invasive bladder cancer;

^a^Negative cytology from our cohorts includes atypical urothelial cells and suspicious urothelial cancer cells

To determine the analytical sensitivity, we analysed expression of later 3 molecules (CD24, CD49f, and NANOG) in 17 primary UCB tissues with matched urine samples. The expression levels of these three molecules (CD24, CD49f, and NANOG) in primary tumour tissues were significantly higher in subjects with positive expression in urine samples than in those with negative urine expression (Supplementary Fig. S2). The concordance rate between primary tumours and the matched urine samples (analytical sensitivity) was 77.8% (7/9) for CD24, 70.0% (7/10) for CD49f, and 64.3% (9/14) for NANOG (Fig. 4e). The clinical sensitivity (detection of cancer by urine test of these three genes) of CD24, CD49f, and NANOG were 47.1% (8/17), 52.93% (9/17), and 52.93% (9/17), respectively (Fig. 4e). At least one of the three genes was overexpressed in primary tumours and urines of all the samples analysed. Based on reasonable analytical and clinical sensitivity in the training cohort, a combination panel of these three CSC-related molecules may have potential to detect UCB with high sensitivity and specificity using clinical samples.

### Validation of a panel of three CSC-related genes (CD24, CD49f, and NANOG) in an independent cohort of urine sample for early detection of UCB

To confirm the detection accuracy of combination of three CSC-related molecules (CD24, CD49f, and NANOG), we tested an independent validation cohort consisting of urine samples from 60 UCB and 183 control subjects (Table 1). Again, higher expression levels of these three molecules were observed in the urine samples from UCB subjects than controls (Supplementary Fig. S3). Using the same cut-off as of training cohort, the individual sensitivity and specificity for cancer detection were 35.0% (21/60) and 91.3% (167/183) for CD24, 35.0% (21/60) and 83.6% (153/183) for CD49f, and 51.7% (31/60) and 88.5% (162/183) for NANOG, respectively (Table 2). The combination panel of these three genes yielded an overall sensitivity of 81.7% (49/60) and specificity of 74.3% (136/183) (Fig. 4d). Of note, this combination panel (CD24, CD49f, and NANOG) detected NMIBC with a sensitivity of 80.9% (38/47) and low-grade UCB with a sensitivity of 80.0% (12/15) (Table 2). In addition, 14 (82.4%) out of a total of 17 cytology-negative UCB samples were detected by this combination panel.

Finally, we assessed the UCB detection accuracy by three markers (CD24, CD49f, and NANOG) panel urine testing by combining training and validation cohorts (total 84 and 207 urine samples from UCB and control subjects, respectively). This analysis yielded a sensitivity and specificity of 82.1% and 75.8%, respectively (Table 2). The positive and negative likelihood ratio of this test was 3.40 (95% confidence interval (CI), 2.71–4.08) and 0.24 (95% CI, 0.15–0.36), respectively (Supplementary Table. S4).

## Discussion

A growing body of evidence indicates that CSCs are a driving force behind tumour initiation, metastasis, and therapeutic resistance.^10^ Therefore, identification of the molecules responsible for urothelial cancer stemness properties may facilitate the development of novel therapeutic strategies and biomarkers for non-invasive UCB detection. Here we showed that CD24 has an essential role in maintaining the urothelial cancer stem-like traits. In addition, to our knowledge, we are reporting for the first time that CSC-related molecules have potential as clinically useful urinary biomarkers for non-invasive detection of UCB.

CD24 is a lynchpin of tumorigenesis and metastatic progression in UCB.^13,15,16^ However, the relevance of CD24 in urothelial cancer stem-like traits remains unclear. In this study, for the first time we characterized CD24 as a major determinant of urothelial stemness, supporting previous findings that CD24-expressing cells exhibit aggressive phenotype. Although we genetically inhibited CD24 using a lentiviral-based approach that may not be suitable for clinical use, Overdevest et al.^15^ demonstrated that treatment with an anti-CD24 monoclonal antibody led to reduced tumour growth and metastasis, resulting in prolonged survival in UCB xenograft model. Collectively, our pre-clinical data suggests that CD24 could be a promising therapeutic target to efficiently eliminate urothelial CSCs.

The exact molecular mechanisms for CSC generation and maintenance via CD24 are incompletely understood. We observed the downregulation of several CSC-related molecules such as CD133, ABCG2, and YAP1 in CD24-sh cells and expression level of these molecules were higher in high-CD24-expressing cells, indicating a potential crosstalk between CD24 and these CSC-related molecules. CD133, a pentaspan transmembrane glycoprotein, has been used as a surface marker for isolation of urothelial CSCs.^11,25^ ABCG2 is a drug transporter and this molecule actively effluxes varieties of chemotherapeutic agents that may provide CSCs with a selective survival advantage against chemotherapy.^26^ YAP1 is a downstream transcription effector of the Hippo pathway^27^ and we recently demonstrated that urothelial cancer stem-like traits are driven by the YAP1-SOX2 signalling axis that is an upstream regulator of CD24 expression.^11^ CD24 interacts with Src to promote its kinase activity^17^ and activated Src has been implicated in regulating YAP1.^11,28^ Thus, the regulatory circuitry between YAP1 and CD24 may accelerate urothelial CSC maintenance and progression. Further research is needed to elucidate these complex crosstalk mechanisms.

Urine cytology analysis is a non-invasive approach for cancer detection with a high specificity ( > 95%),^29^ but it is limited by its low sensitivity (35–55%),^5^ especially for low-grade ( < 20%) and low-stage ( < 40%) disease.^6,7^ Sensitivity is generally considered more important than specificity for screening and surveillance, as the missing of early disease increases the risk of progression to advanced disease and a poor clinical outcome.^30^ Although several urine-based diagnostic assays have been approved by the U.S. Food and Drug Administration,^5,31,32^ these assays do not overcome the low sensitivity for low-grade disease. Therefore, improvement of the detection sensitivity for low-stage and low-grade disease is one of the central goals of urine-based tests. In this study, the combination panel (CD24, CD49f, and NANOG) yielded high sensitivity for cancer detection not only for NMIBC (80.9%), but also for low-grade UCB (80.0%). Most importantly, 82.4% of UCB specimens negative by cytology were positive by CSC marker test in urine. Therefore, this CSC-related panel with high sensitivity could provide a valuable adjunct to urine cytology for UCB detection, if confirmed by a larger prospective study.

CD49f mediates the stem cell niche via interactions with the extracellular matrix and communication between tumour cells and the tumour microenvironment.^33^ In UCB, CD49f is downregulated during differentiation^34^ and has been utilized to enrich CSC population.^35^ NANOG is a key pluripotent transcription factor^36^ and predominantly expressed in urothelial CSCs.^37^ Thus, all the three molecules (CD24, CD49f, and NANOG) act as biologically relevant CSC factors in UCB. As we did not observe concordant changes in CD49f and NANOG expression due to alteration of CD24 expression in CD24-sh cells and CD24-expressing cells, the combination panel may be able to detect different CSC populations and each of these molecules could be an independent marker for this heterogeneous disease. In fact, 39 of 60 UCB urine samples in the validation cohort showed negative expression of CD24, and 9 (23.1%) and 21 (53.8%) out of 39 urine samples without CD24 expression showed positive for CD49f and NANOG, respectively. This observation also supports the multi-clonal origin of this heterogeneous disease.^38^ Although its needs further validation, some inconsistency of findings between primary tumours and urine may be due to the site of the primary tumours that were analysed. It could happen that we analysed the tumour site that did not shed the tumour cells in the urine and the source of tumour cell may be other sites of the same bladder.

Several studies reported promising panels of urinary mRNAs determined by qRT-PCR for UCB detection, including the commercially available Cxbladder assay ^39^^-^^42^. Compared with these assays, our overall sensitivity of our assay was similar, but specificity was relatively low, partially due to the use of different reference genes and selection approaches. Cxbladder adapted CXCR2 as a reference gene to reduce the false positive rate.^39^ Other assays considered genes that are expressed stably and with little variability in exfoliated urinary cells.^40–42^ Thus, the use of suitable reference genes may improve the specificity of our as~~say. In addition, the novel transcriptome profiling approach may yield more sensitive and specific CSC-related biomarkers in urine.^43^

Study limitations include the relatively small sample size and possible bias due to retrospective analysis and empirical selection of CSC-related molecules for the urinary biomarker. In addition, our cohort may not be representative of the general population at risk for UCB because of the lack of relevant clinical data, including smoking history, occupational exposure history, and patient outcome. Infections or any other inflammatory disease in the urinary tract may influence expression of these biomarkers. Therefore, although promising, our findings cannot be considered conclusive, and extensive validation is needed in a larger independent cohort including various urologic conditions to assess the clinical utility of combination panel of three CSC-related molecules.

In summary, we demonstrated that CD24 drives cancer stem-like traits and serves as a promising non-invasive urinary biomarker for UCB detection. In addition, we also identified a panel of CSC-related molecules that has potential as a urinary biomarker for UCB detection with a high sensitivity and specificity. These findings may facilitate the development of improved therapeutic strategies and non-invasive detection of UCB.

### Availability of data and material

The datasets generated during and/or analysed during the current study are available from the corresponding author on reasonable request.

## Electronic supplementary material

Supplementary Figures

Supplementary Table S1

Supplementary Table S2

Supplementary Table S3

Supplementary Table S4
