# Supplementary material for: CD24 regulates cancer stem cell (CSC)-like traits and a panel of CSC-related molecules serves as a non-invasive urinary biomarker for the detection of bladder cancer
Source: Br J Cancer. 2018 Oct 17;119(8):961–70. doi: 10.1038/s41416-018-0291-7 (PMC6203855; doi:10.1038/s41416-018-0291-7)
Supplement: Supplementary file 1 — Supplementary Figures [file 41416_2018_291_MOESM1_ESM.docx]

**
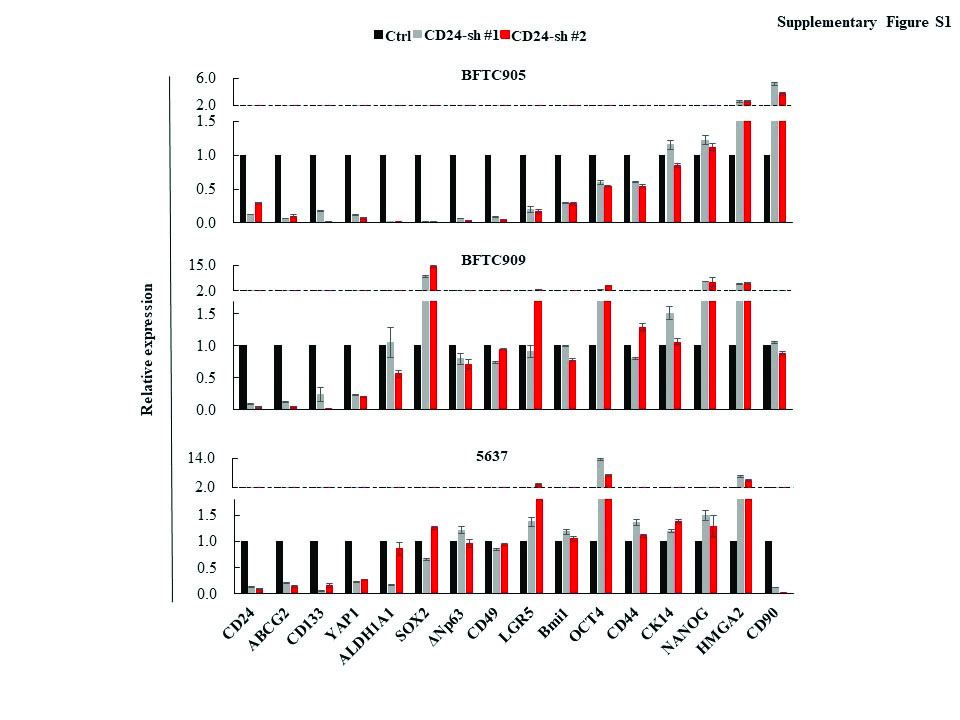
**

**Supplemental Figure S1.**

CSC-related molecules modulated by CD24 knockdown in BFTC 905, BFTC 909, and 5637 cell lines. The relative mRNA expression levels of 15 potential CSC-related molecules in CD24-sh cells were calculated considering the expression values equal to 1.0 in CD24-Ctrl cells, as measured by qRT-PCR and normalized by β-actin. The expression levels of ABCG2, CD133, and YAP1 were consistently downregulated due to knockdown of CD24. Data are from three independent experiments. Each error bar indicates mean ± SEM.


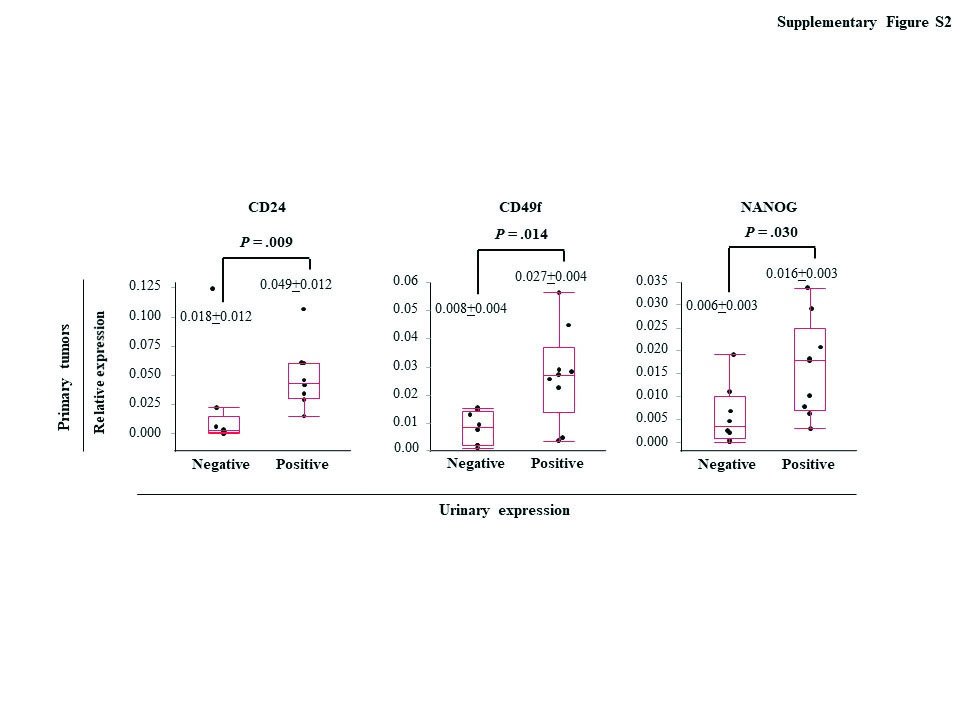


**Supplemental Figure S2.**

The relative expression levels of CD24, CD49f, and NANOG in primary tumor tissues according to their expression status in the matched urine samples. Each data indicates mean ± SEM.　Wilcoxon–Mann–Whitney test were performed.


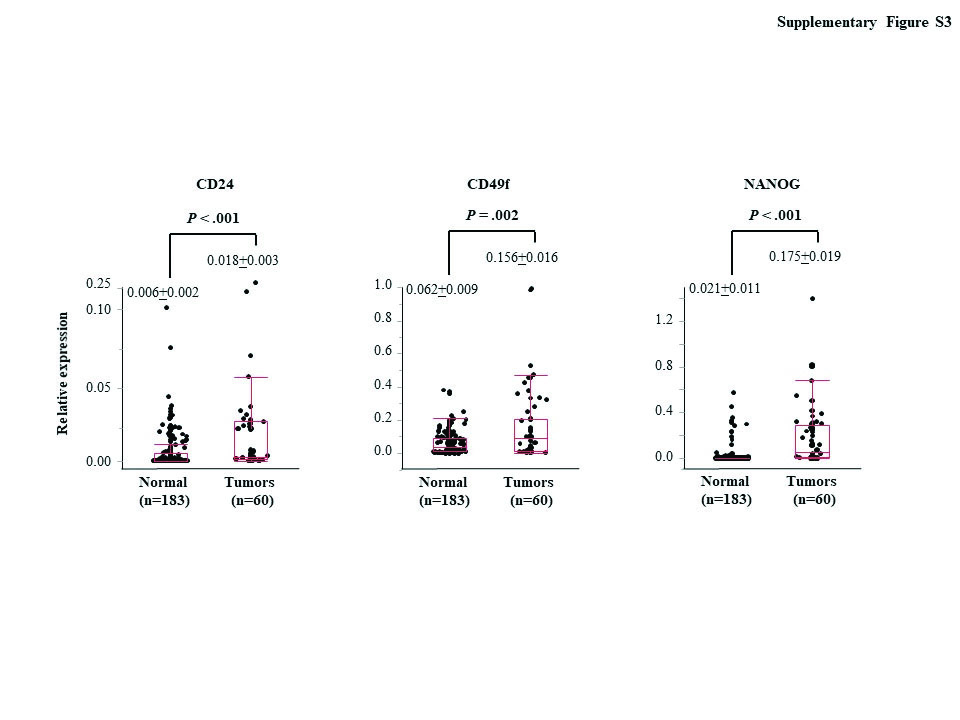


**Supplemental Figure S3.**

Box plots of the relative expression levels of CD24, CD49f, and NANOG in urine samples from UCB (n=60) and control (n=183) in an independent validation cohort. Data indicates mean ± SEM. Wilcoxon–Mann–Whitney test was performed.
